# Quality and readability of online patient education materials for trigeminal Neuralgia: A cross-sectional review

**DOI:** 10.1016/j.bas.2026.107476

**Published:** 2026-10-15

**Authors:** Philbert Yu Xuan Teo, Selnan Wuyep, Andreas K. Demetriades

**Affiliations:** a Edinburgh Medical School, The University of Edinburgh, Edinburgh, United Kingdom; b Department of Neurosurgery, Leiden University Medical Centre, Leiden, the Netherlands; c Department of Neurosurgery, Royal Infirmary Edinburgh, NHS Lothian, Edinburgh, United Kingdom

**Keywords:** Trigeminal neuralgia, Patient education, Readability, DISCERN, Internet

## Abstract

**Background/objectives:**

Patients with trigeminal neuralgia frequently seek online information about symptoms, diagnosis and treatment options. Previous work has identified weaknesses in the quality of online trigeminal neuralgia information and high estimated reading demands. This study evaluated the quality and readability of online patient education materials on trigeminal neuralgia.

**Methods:**

Google was searched for the term “trigeminal neuralgia,” and the first 30 results were screened, assessing the linked webpage rather than the parent website. English-language, freely accessible, patient-facing webpages were included. Two reviewers independently assessed quality using the DISCERN instrument, and item-level scores were averaged for analysis. Readability was assessed using standard readability formulae from ReadabilityFormulas.com after removal of non-educational webpage content.

**Results:**

Of 30 screened results, 22 webpages were included. Included webpages comprised 10 general health information sites and 12 healthcare provider sites. The mean Total DISCERN score across reviewers was 49.32 ± 9.43, and the mean overall DISCERN rating was 3.30 ± 0.70. The lowest mean item scores concerned the consequences of no treatment (1.61), effects on quality of life (1.77), and clarity of evidence sources (2.14). The mean average reading level consensus was 10.93 ± 1.49, with 21 webpages (95.5%) exceeding grade 8 by this measure and none at or below grade 6.

**Conclusions:**

The sampled online patient education materials on trigeminal neuralgia were commonly written above recommended reading levels and varied in DISCERN-assessed information quality. Clinicians should be aware that patients may encounter resources that are difficult to read or incomplete. Clinical accuracy, patient comprehension, and effects on treatment decisions were not assessed.

## Highlights

• Online patient education materials for trigeminal neuralgia are frequently written at reading levels above recommended guidelines, and the vast majority (95.2%) exceed the eighth-grade level, potentially limiting patient comprehension.
• The quality of online trigeminal neuralgia information, assessed using the DISCERN instrument, is moderate and variable with notable deficiencies in citing sources, discussing treatment risks, and describing the consequences of no treatment.
• While many online resources provide relevant information, they consistently lack support for shared decision-making and fail to address the effects of treatment on quality of life, highlighting gaps in patient-centred content.
• Given the complexity of treatment decisions in trigeminal neuralgia, healthcare providers should be aware that patients may encounter online materials that are both difficult to read and incomplete; efforts are needed to improve the accessibility and quality of patient-facing resources.

## 1 Introduction

Trigeminal neuralgia (TN) is a severe facial pain disorder characterised by recurrent paroxysms of unilateral facial pain. Treatment decisions may involve long-term pharmacological therapy, which may be associated with adverse effects, or procedural interventions such as percutaneous procedures, stereotactic radiosurgery, and microvascular decompression. These options vary in invasiveness, durability, risks, and suitability. Patient information therefore needs to be both reliable and understandable (Bendtsen et al., 2019).

Patients commonly use online resources before or after clinical consultation, and online health information can influence consultation behaviour, expectations, and health-related decisions (Thapa et al., 2021). In TN, this may shape understanding as well as expectations about diagnosis, treatment choice, risk, and prognosis. In 2014, we evaluated online information on trigeminal neuralgia using the DISCERN instrument and identified weaknesses in balance, sourcing, discussion of treatment risks, and description of the consequences of no treatment (Demetriades et al., 2014).

Readability is a related but distinct issue. A webpage may be broadly accurate yet still be written at a reading level that limits accessibility for many patients. Health literacy guidance commonly recommends that patient-facing written materials be pitched below average adult reading level. US health literacy guidance from the Agency for Healthcare Research and Quality Health Literacy Universal Precautions Toolkit notes that the average adult reads at approximately eighth to ninth grade level and recommends assessing written materials through several approaches, including patient feedback (Agency for Healthcare Research and Quality). Recent readability work in anterior cervical discectomy and fusion patient education materials has shown that online materials may remain above recommended reading levels (Sedani et al., 2025). Joseph et al. assessed TN-specific readability in 2020 using seven formulae and found that most sampled webpages exceeded an eighth-grade reading level (Joseph et al., 2020).

Accordingly, this study aimed to assess the quality and readability of current online patient education materials on trigeminal neuralgia and to identify weaknesses in treatment information and estimated reading demands. We considered these resources as general patient education and preparation for consultation. We did not evaluate them as validated decision aids or test whether they met individual patients’ information needs.

## 2 Materials and methods

### 2.1 Study design and search strategy

This was a descriptive cross-sectional review of publicly available online patient education materials. Google.com was searched in private browsing mode using the term "trigeminal neuralgia" on 8 April 2026. The search was performed from Edinburgh, UK, with no user logged into a Google account.

The first 30 results were screened. This approach mirrored the sampling strategy used in the previous trigeminal neuralgia internet review and reflects evidence that users tend to focus on early search results when seeking health information (Demetriades et al., 2014; Eysenbach and Köhler, 2002). In the earlier TN review's preliminary outpatient survey, only 30% looked beyond the second search page and none beyond the third (Demetriades et al., 2014). Eysenbach and Köhler found that 97.2% of observed health-search clicks were within the first 10 results (Eysenbach and Köhler, 2002). The search therefore reflects early Google results rather than a representative sample of all online TN information. Supplementary Table S1 records the retrieved URLs and screening decisions. Search date, browser setting, location, and login status were recorded in the analysis workbook.

### 2.2 Eligibility criteria

Eligible webpages were English-language, freely accessible, patient-facing resources containing educational information on trigeminal neuralgia. Exclusion criteria were duplicate links, inaccessible pages, professional-facing resources, peer-reviewed journal articles, videos without substantial accompanying text, discussion forums, social media posts, news articles, pages consisting predominantly of advertisements, and pages containing insufficient educational text for meaningful quality or readability assessment. Professional-facing resources were defined as webpages primarily intended for clinicians or researchers rather than patients or the general public. Pages with insufficient educational text were defined as pages containing only brief summaries, listings, or promotional material without substantive explanatory content. We only assessed the individual retrieved webpage or search result, rather than the parent website as a whole. Other links found within the webpage accessed from our search, such as linked pages or booklets, were not assessed unless they were themselves returned among the sampled results.

### 2.3 Source classification

Source classification was performed by the study team according to the primary organisational identity and purpose of each website. For descriptive analysis, we grouped hospital, clinic and other treatment-provider pages as healthcare provider resources. The second group comprised general health information resources, including government agencies, professional associations, charities and other information publishers. These groups describe organisational function; they do not establish ownership, profit status, commercial intent or freedom from bias. Group comparisons were treated as exploratory because of small category sizes.

We also recorded the responsible publisher or organisation country and visible publication, update or review dates from the archived pages. Country describes the publisher's location, not the webpage's geographic availability or intended audience.

### 2.4 Quality assessment

Quality was assessed using the DISCERN instrument, a 16-item tool for evaluating written consumer health information on treatment choices. Items 1 to 8 assess reliability, items 9 to 15 assess treatment information, and item 16 provides an overall quality rating (Charnock et al., 1999). Each DISCERN item is scored from 1 to 5. For items 1 to 15, 1 indicates that the criterion is not fulfilled and 5 that it is fulfilled, with intermediate scores indicating partial fulfilment. Item 16 is a separate overall-quality judgement. We report this rating separately and also report the sum of all 16 items, with a possible range of 16-80. DISCERN does not independently verify clinical accuracy, currency or disease-specific completeness (Charnock et al., 1999; Charnock, 1998). P.Y.X.T. and S.W. were both medical students at the time of scoring; S.W. is now a resident physician. The assessment did not include any other physician or patient assessor. For descriptive interpretation, total scores were grouped as very poor, 16 to <27; poor, 27 to <39; medium, 39 to <51; good, 51 to <63; and excellent, 63–80. Continuous intervals retain half-point values created by averaging the two reviewers. These labels describe the adopted scoring convention and are reported alongside the ungrouped scores.

Two reviewers independently scored all included webpages. Reviewers used the published DISCERN guidance when scoring webpages. For analysis, item-level scores were averaged across reviewers. Absolute differences in total DISCERN scores were calculated descriptively to assess reviewer variability.

### 2.5 Readability assessment

For each included webpage, patient-facing educational text was extracted into plain-text format. Plain text was used for readability analysis only, while DISCERN ratings concerned the original webpage. Non-educational material was removed before analysis, including navigation menus, copyright notices, disclaimers, author information, hyperlinks, references, figure captions, addresses, and telephone numbers. The cleaned text was then analysed using ReadabilityFormulas.com, consistent with prior readability studies of online patient education materials (Sedani et al., 2025). Recorded metrics comprised Flesch Reading Ease (FRE), Flesch-Kincaid Grade Level (FKGL), Gunning Fog (GF), Coleman-Liau Index (CLI), Simple Measure of Gobbledygook (SMOG) Index, Automated Readability Index (ARI), Linsear Write Formula (LWF), and the average reading level consensus. These seven named formula outputs and the calculator-reported Average Reading Level Consensus (ARLC) were used. ARLC is a software-reported summary, not an eighth independent formula.

FRE and FKGL are calculations that utilise sentence length and syllables per word to determine readability. GF is calculated using the number of words with three or more syllables (polysyllabic) and average length of sentence. CLI is calculated using average sentence length and average number of letters per word but does not assess number of syllables in a text. SMOG index extracts the number of polysyllabic words across three ten-sentence samples from the beginning, middle and end of a text, for calculations (Joseph et al., 2020; Kher et al., 2017). ARI and LWF in similar fashions are also based on calculations including sentence length and other text characteristics to determine readability (Joseph et al., 2020). Lower scores in all formulae here except FRE indicate easier texts or better readability. FRE uses a different scale, with higher scores indicating easier text. Conventional interpretation bands are 90–100, very easy; 80 to <90, easy; 70 to <80, fairly easy; 60 to <70, standard; 50 to <60, fairly difficult; 30 to <50, difficult; and <30, very difficult (ReadabilityFormulas.com). These bands are not mathematical bounds of the formula.

The six grade-level formulae estimate years of United States schooling. Lower values indicate lower estimated reading demands, and grade 12 corresponds approximately to the end of secondary school. We used grades 6 and 8 as descriptive reference points. These cutoffs do not establish whether an individual patient can understand a page but reflect the currently established reading difficulty levels, likely to influence the ease by which a patient can access written information.

### 2.6 Statistical analysis

Data management and descriptive analyses were performed in Microsoft Excel. Continuous variables are reported as mean ± standard deviation, median, minimum, and maximum, and categorical variables as counts and percentages. DISCERN scores from the two reviewers were averaged before analysis. Reviewer variability was summarised descriptively using absolute differences in total DISCERN scores. No inferential statistical testing was performed because the study was descriptive, and subgroup sizes were small. As a post hoc sensitivity analysis, we repeated the headline DISCERN and readability summaries after excluding the retrospectively reassessed NHS treatment page at search rank 10.

## 3 Results

### 3.1 Search yield

The screening record contains 30 search results. On reassessment against the stated eligibility criteria, the distinct NHS treatment page at rank 10 was considered eligible and included in the revised analysis. The TNA homepage at rank 12 and Brain & Spine Foundation landing page at rank 26 remained excluded because the retrieved pages themselves contained insufficient educational text. The separate Face Pain booklet linked from the rank-26 page was outside the webpage-level assessment rule. The final screening decision therefore comprised 22 included webpages and eight exclusions: six professional or scholarly resources and two pages with insufficient educational text at the retrieved URL. Included webpages comprised 12 healthcare provider pages and 10 general health information pages (Table 1). Publisher organisations were evenly split between the United Kingdom and United States (11 each).

**Table 1:** Search yield and revised source groups.

Measure	n	%
Search results screened	30	100.0
Included webpages	22	73.3
Excluded results	8	26.7
Healthcare provider pages	12	54.5
General health information pages	10	45.5
Publisher organisation: United Kingdom	11	50.0
Publisher organisation: United States	11	50.0

Among the 21 included pages with an original 8 April capture date, 13 (61.9%) displayed a publication, update or review date and three also displayed a planned next-review date. Eight (38.1%) did not display such a page date. The original PDF for the newly included NHS treatment page had not been retained. It was reassessed from a 27 September 2026 print of the same URL, which displayed “Page last reviewed: 27 January 2023” and “Next review due: 27 January 2026”; these later-captured fields were not counted in the 8 April totals.

### 3.2 Readability

The mean reported reading-level consensus was 10.93 ± 1.49 (median 10.94; range 7.84–13.49). One page (4.5%) had a consensus reading level below grade 8 (7.84), and none were at or below grade 6, therefore 21 of 22 pages (95.5%) exceeded grade 8. Mean FRE was 49.55 ± 7.81 (Table 2). Individual formulae differed: the number of pages at or below grade 8 ranged from zero to four, and LWF classified one page at or below grade 6.

**Table 2:** Readability outputs.

Output	Mean ± SD	Median	Observed range
Average reading level consensus	10.93 ± 1.49	10.94	7.84–13.49
Flesch Reading Ease	49.55 ± 7.81	50.00	36.00–63.00
Flesch-Kincaid Grade Level	10.13 ± 2.03	9.78	6.54–13.75
Gunning Fog	12.20 ± 1.49	12.20	8.70–14.70
Coleman-Liau Index	11.82 ± 1.31	11.60	9.21–14.50
SMOG Index	9.82 ± 1.61	9.68	6.71–12.85
Automated Readability Index	10.58 ± 2.41	10.26	6.30–15.07
Linsear Write Formula	11.20 ± 2.54	11.01	5.44–15.77

All outputs except FRE express estimated US grade level. The consensus is the calculator's recorded summary output.

### 3.3 DISCERN assessment

The mean total DISCERN score was 49.32 ± 9.43 (median 52.25; range 28.50–62.00), and the mean overall rating was 3.30 ± 0.70 (Table 3). Under the descriptive categories specified above, 13 pages (59.1%) fell in the good category, five (22.7%) in medium and four (18.2%) in poor. None fell in the excellent or very poor categories. The mean absolute difference between reviewer totals was 7.00 ± 5.21.

**Table 3:** DISCERN summary measures.

Measure	Mean ± SD	Median	Observed range
Reviewer 1 total	50.55 ± 9.87	52.50	30.00–64.00
Reviewer 2 total	48.09 ± 10.80	48.50	27.00–65.00
Mean total across reviewers	49.32 ± 9.43	52.25	28.50–62.00
Mean overall rating	3.30 ± 0.70	3.50	2.00–4.50
Absolute reviewer difference	7.00 ± 5.21	5.50	1.00–18.00

Relevance remained the highest-scoring item (4.55), followed by achievement of stated aims (4.11) and presentation of more than one treatment option (4.07). All 22 webpages had an averaged relevance score of at least 4, and 20 of 22 (90.9%) scored at least 4 for achieving their stated aims. In contrast, 19 of 22 (86.4%) had averaged scores of 2 or less for the consequences of no treatment, 17 of 22 (77.3%) for effects on quality of life, and 13 of 22 (59.1%) for clarity of information sources. The final mean score for dating of information was 2.59.

### 3.4 Source groups

Healthcare provider pages had a mean total DISCERN score of 47.58 ± 10.24 (n = 12), compared with 51.40 ± 8.40 for general health information pages (n = 10) (see Table 4). Mean reported reading-level consensus values were 11.19 ± 1.42 and 10.62 ± 1.58, respectively; mean FRE values were 48.08 ± 6.97 and 51.30 ± 8.74 (Table 5).

**Table 4:** Mean DISCERN item scores.

Item	Mean score
1. Aims clear	3.95
2. Achieves aims	4.11
3. Relevant	4.55
4. Sources clear	2.14
5. Date of information clear	2.59
6. Balanced and unbiased	3.39
7. Additional sources and support	2.80
8. Areas of uncertainty	2.68
9. How treatment works	3.50
10. Treatment benefits	3.39
11. Treatment risks	2.98
12. What happens without treatment	1.61
13. Effects on quality of life	1.77
14. More than one treatment option	4.07
15. Support for shared decision-making	2.50
16. Overall quality	3.30

**Table 5:** Descriptive results by revised source group.

Source group	n	Total DISCERN	Consensus grade	Flesch Ease
Healthcare provider	12	47.58 ± 10.24	11.19 ± 1.42	48.08 ± 6.97
General health information	10	51.40 ± 8.40	10.62 ± 1.58	51.30 ± 8.74

In the post hoc sensitivity analysis excluding the retrospectively reassessed NHS treatment page, the mean total DISCERN score was 48.83, the mean reported reading-level consensus was 10.81, and 20 of 21 webpages remained above grade 8. The headline interpretation was therefore unchanged.

## 4 Discussion

This cross-sectional audit of online patient education materials on trigeminal neuralgia identified two main issues: high reading levels and variable quality of treatment information. Many included pages were produced by recognisable healthcare providers or general health information sources, but the average reading level remained above the sixth to eighth grade level commonly used as a target for patient-facing health information (Agency for Healthcare Research and Quality; Daraz et al., 2018). The mean reported consensus was 10.93, and 21 of 22 webpages exceeded grade 8. These findings describe the sampled written resources and do not establish that the medical advice was inaccurate or that patients could not understand it.

The readability findings are clinically relevant as most included webpages were written above commonly recommended reading levels for patient-facing health information. This may reduce accessibility for patients seeking to understand symptoms, treatment options, and associated risks. These findings align with broader evidence that online health information is frequently written above recommended public reading levels (Daraz et al., 2018).

The lowest DISCERN scores related to the consequences of no treatment, effects on quality of life and identification of information sources, while achievement of aims and presentation of more than one treatment option were relative strengths. Online information may inform choices between undergoing medical and surgical treatment, each with different considerations like adverse effects and patient preference, further emphasising the potential clinical impact of these findings. These findings identify specific domains for revision but do not establish clinical accuracy, exhaustive disease-specific information or adequacy for individualised treatment decisions.

### 4.1 Comparison with earlier TN studies

The 2014 review assessed 24 of the first 30 Google search results after excluding six published articles or repetitions (Demetriades et al., 2014). It reported that 71% of assessed websites did not clearly identify information sources, 79% did not describe the consequences of no treatment and 79% did not describe treatment effects on quality of life. Those same domains remain among the weakest in the present sample. Conversely, the earlier review found that 95% of sites presented more than one treatment option, which was also a relative strength in the present study. In 2014, 34% of sites did not clearly state when information was compiled or updated. In our current study, eight of 22 sites (36.4%) lacked clearly displayed publication, update or review dates in the captured pages.

The earlier review also described a commercial Gamma Knife treatment centre that promoted this treatment modality without balanced evidence (Demetriades et al., 2014). No Gamma Knife-specific page appeared as a separate result among the first 30 Google results in the 2026 sample. This reflects a difference in the composition of the sampled search results and should not be interpreted as evidence that information about, or clinical use of, stereotactic radiosurgery has declined.

TN-specific readability has also been studied previously by Joseph et al.: they assessed 13 patient-facing TN webpages with the same seven readability formulae and reported mean scores of 10.9 for FKGL, 15.0 for GF, 10.9 for SMOG, 12.1 for CLI, 11.9 for Automated Readability Index and 12.4 for LWF, with a mean FRE score of 42.1 (Joseph et al., 2020). The corresponding values in the present sample were 10.13, 12.20, 9.82, 11.82, 10.58, 11.20 and 49.55, respectively. The observed results in comparison to the earlier study show a decrease in 6 of the readability formulae alongside an increase in the mean FRE score. This may point towards an improvement in the readability of the currently available online materials and may represent progress in making online patients’ information more accessible, with easier read texts.

This study updates earlier work by reassessing online trigeminal neuralgia information using a comparable sampling approach while also incorporating readability analysis. This combined assessment is important because content quality and readability are potential barriers to patient understanding: a resource may be accurate but difficult to read, or easy to read but incomplete in its discussion of risks, uncertainty or alternative treatment options. In this study, information quality and readability were assessed in the same sample, with individual DISCERN component findings identifying specific areas for revision.

### 4.2 Clinical implications

Clinicians should be aware that patients seeking information on trigeminal neuralgia online may encounter material that is difficult to read, incomplete, or inconsistently balanced. Previous studies have highlighted that online written medical information may be communicated at reading levels higher than that of the general public (Kher et al., 2017). Healthcare organisations and patient-information providers should aim to improve readability while maintaining clear discussion of treatment options, risks, benefits, uncertainty, and the consequences of no treatment. The effect on quality of life and the role of patients’ preferences in making decisions should also be covered more clearly.

Although our study did not directly assess the comprehensiveness of TN specific information that was provided, it is also important that online content provides patients with information such as; explanations on why recommendations can differ depending on disease subtype, imaging, previous treatment and recurrent symptoms. It should explain how well medications work and their side effects, the risks and limitations of procedures, and how patient preferences may affect treatment choices (Bendtsen et al., 2019). Our study did not assess how completely each resource covered all of these TN-specific issues.

Patient feedback and user testing should guide revisions to the material and assess whether the resulting information answers patients’ questions (Agency for Healthcare Research and Quality). Lower formula scores alone cannot establish better comprehension. Page-specific findings should be shared with the relevant information providers to inform revisions, while any subsequent improvement in patient understanding or usefulness would need to be evaluated by involving patients.

### 4.3 Strengths and limitations

Strengths of this study include a reproducible search strategy, use of a sampling frame comparable to that of a previous trigeminal neuralgia internet review, independent dual-reviewer DISCERN assessment, and readability analysis of cleaned patient-facing text. The study also retained excluded results, supporting transparency of screening.

Limitations include use of a single search engine and search term, restriction to English-language written materials, exclusion of non-text formats such as videos, and the dynamic nature of search engine results, which may vary by time, location and user context. The findings therefore apply to the webpages assessed and should not be generalised to all online TN information. Nevertheless, our findings serve as useful insight for website authors on important elements to include when developing or updating online patient education materials. It is aimed that as online information grows with time our study may also, as we have done (Demetriades et al., 2014; Joseph et al., 2020; Kher et al., 2017), be used to compare and assess for improvements in online education resources.

DISCERN assesses the quality of written treatment information but does not establish whether the medical information is accurate or fully up to date. We did not check every medical statement for accuracy or assess whether each webpage was complete for different clinical situations. No patients took part in the assessment and we did not conduct user testing, so the study does not show whether patients understood the information, found it useful, or if it addressed patients’ priorities when making treatment decisions.

In addition, readability formulae are imperfect proxies for patient comprehension and do not account for layout, numeracy, images, prior knowledge or broader health literacy. We did not formally assess WCAG compliance, and we did not treat publisher accessibility statements as evidence of compliance (World Wide Web Consortium). The original cleaned text and calculator settings were not retained for every webpage, which limits exact reproduction of the original readability results.

The individual webpage returned in the search was assessed rather than the website as a whole. Some organisations provide information across several linked pages or separate booklets, so assessing the whole website could produce different DISCERN scores and would require a predefined rule for which material to include.

## 5 Conclusion

Although we have highlighted some potential progress in the readability of available online education materials on TN, the sampled resources still remain written above recommended readability levels. The webpages also varied in DISCERN-assessed information quality. Improvement efforts for patient-facing resources should address both the quality of treatment information and the accessibility of language, particularly given the complexity and consequences of treatment decisions in trigeminal neuralgia. The weakest domains concerned the consequences of no treatment, quality-of-life effects and identification of evidence sources. Clinical accuracy, suitability for individual treatment decisions and benefits to patients require separate assessment.

## Informed consent statement:

Not applicable.

## Informed consent statement

Not applicable.

## Author contributions

Conceptualisation, A.K.D. and P.Y.X.T.; methodology, P.Y.X.T. and A.K.D.; validation, S.W. and A.K.D.; formal analysis, P.Y.X.T.; investigation, P.Y.X.T. and S.W.; data curation, P.Y.X.T.; writing the original draft, P.Y.X.T.; reviewing and editing, S.W. and A.K.D.; supervision and project administration, A.K.D.

## Ethics and consent:

Not applicable. The study analysed publicly available information and involved no patient participants or identifiable patient data.

## Institutional review board statement:

Not applicable. This study analysed publicly available online patient education materials and did not involve human participants, animals, or patient-identifiable data.

## Institutional review board statement

Not applicable. This study analysed publicly available online patient education materials and did not involve human participants, animals, or patient-identifiable data.

## Data availability statement:

The data supporting the findings of this study are available in the Supplementary material.

## Funding:

This research received no funding.

## Conflicts of interest:

The authors declare no conflict of interest.

## Appendix A Supplementary data

Supplementary Table S1 provides the screening record, source classification, readability outputs and both reviewers' item-level DISCERN ratings.
